# Research and Application Progress of Geopolymers in Adsorption: A Review

**DOI:** 10.3390/nano12173002

**Published:** 2022-08-30

**Authors:** Jinyun Xu, Minjing Li, Di Zhao, Guoqiang Zhong, Yu Sun, Xudong Hu, Jiefang Sun, Xiaoyun Li, Wenju Zhu, Ming Li, Ziqi Zhang, Yu Zhang, Liping Zhao, Chunming Zheng, Xiaohong Sun

**Affiliations:** 1 Tianjin Key Laboratory of Green Chemical Technology and Process Engineering, State Key Laboratory of Separation Membrane and Membrane Processes, School of Chemical Engineering, Tiangong University, Tianjin 300387, China; 2 Key Laboratory of Advanced Ceramics and Machining Technology of the Ministry of Education, School of Materials Science and Engineering, Tianjin University, Tianjin 300072, China; 3 Beijing Key Laboratory of Diagnostic and Traceability Technologies for Food Poisoning, Beijing Center for Disease Prevention and Control, Beijing 100013, China; 4 Advanced Materials Research Laboratory, CNOOC Tianjin Chemical Research and Design Institute, Tianjin 300131, China

**Keywords:** geopolymers, adsorption, heavy metals, dyes, wastewater, geopolymer membrane

## Abstract

Geopolymer is a porous inorganic material with a three-dimensional mesh structure, good mechanical properties, a simple preparation process (no sintering) and a low economic cost, and it is environmentally friendly. Geopolymer concrete has been widely used in the construction field, and many other studies have revealed that geopolymer will become one of the most promising inorganic materials with unique structure and properties. This paper provides a review of the development and current status of geopolymers and briefly explains the effects of material proportioning, experimental factors and activators on geopolymer performance. Because of the advantages of high specific surface area and high porosity, geopolymers could be used as adsorbent materials. This paper summarizes the research progresses of the adsorption of metal cations, anions, dyes, and gases by geopolymers, which emphasizes the geopolymer membranes in adsorption, and discusses the challenges and opportunities for the development of more efficient, sustainable and practical adsorption protocols.

## 1. Introduction

Research on the preparation of new environmentally friendly materials using fly ash, metakaolin, and other substances containing large amounts of silicon and aluminum elements as raw materials has become highly popular in recent years. These materials will be processed to produce innovative materials with amorphous and quasi-crystalline three-dimensional mesh structures. This relatively inventive material is referred to as Geopolymer, which was originally conceived by French scientist Joseph Davidovits in 1978 [1,2]. Davidovits [3] prepared for the first time new inorganic silica–alumina gel materials with a three-dimensional structure using the reaction of alkaline solution NaOH/KOH with metakaolin. Developments in geopolymers are not only naturally occurring silicate mineral polymers, but the concept also includes natural minerals, slag, industrial waste, volcanic ash, and solid waste to produce gel polymeric materials with amorphous and quasi-crystalline structures [4,5,6].

With the continuous development of modern technology, porous geopolymers have attracted significant attention from academia and industry under their unique three-dimensional structure. Geopolymer products have stable chemical properties, thermal stability, inexpensive and accessible raw materials, and a simple preparation process [7,8,9,10,11]. The geopolymer has excellent practical applications when used as a modern green inorganic porous material. This research area is surrounded by extensive research directions. The microstructure of geopolymers has more irregular pores with larger specific surface areas and the three-dimensional mesh structure is favorable for capturing adsorbed substances.

Adsorption is a mass transfer process in which a substance is transferred from one phase to another. According to the different forces of attraction between substances, they could be divided into chemisorption and physical adsorption. Contaminants accumulate on the adsorbent surface due to physical forces such as van der Waals forces, hydrogen bonding, hydrophobic interactions, polarity and space forces. The dipole-induced dipole interactions and the chemistry of π–π interactions also produce adsorption effects [12]. Adsorption could be used for purification, decolorization, separation, deodorization, concentration, and detoxification, and it is becoming increasingly important in many areas of production [13]. There are various adsorption materials, and the commonly used inorganic adsorption materials are silica gel, activated alumina, zeolite, etc. Organic adsorbents are polyacrylamide, resin, cellulose, etc. Adsorption as a highly efficient means of pollutant removal technology has received much attention, where adsorption materials directly affect the adsorption effect, and efficient green adsorption materials have become a popular research subject. Researchers have found that nanomaterials exhibit high adsorption capacity for pollutants such as dyes, heavy metal ions, etc. [14,15,16]. With the advancement of technology, new types of adsorbent materials have been reported for the adsorption of pollutants, such as spinel ferrite magnetic materials, nanomagnetite-modified biochar, ligand-immobilized facial composite adsorbent, mesoporous adsorbent, graphene-based adsorbents and agro-industrial waste derived adsorbents [17,18,19,20,21].

Adsorbent materials are green and safe, economical and low-cost, easy-to-recycle, and abundant raw materials. Adsorbent materials may be prepared from minerals, shellfish, starch, tree bark, industrial and agricultural wastes, etc. Adsorbent materials could treat a variety of pollutants such as heavy metal ions, dyes, organic matter, and CO_2_ [22]. Adsorption is possible in combination with other technologies and photocatalytic technology to increase the adsorption capacity while improving the efficiency of dye degradation [23]. However, the preparation process of some adsorbent materials is complicated, the cost is high, and they are not suitable for large-scale use in industrial production, and there are problems such as high environmental requirements, restricted application of single adsorbent, and poor selectivity in practical applications. Compared with other traditional pollutant treatment technologies (electrolysis, photocatalysis, landfill, membrane separation), adsorption has great applicability and economy [24,25]. The use of traditional adsorbents is becoming more and more expensive, the emission of pollutants is strictly managed, and the development and research of economical, efficient and new green adsorbent materials will become the future development trend [26,27,28,29,30].

The geopolymers are porous with stable properties and high adsorption capacity. After modification treatment, the rich properties are expected to compensate for their shortcomings. The low price, abundant raw materials and simple preparation make it highly competitive, and it has potential for widespread use as an adsorbent material. This review will briefly describe the reaction mechanism and current research status of geopolymers, focus on the characteristics of geopolymers and highlight the applications of geopolymers in adsorption.

## 2. Geopolymer Reaction Mechanism and Research Status

### 2.1. Geopolymer Reaction Mechanism

**Figure 1:**
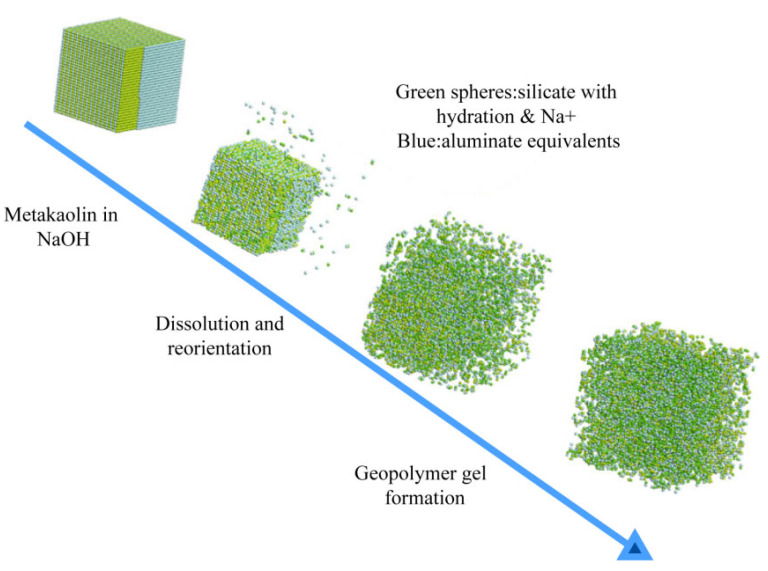
The model demonstrates the gradual formation of a geopolymer gel from solid layered aluminosilicate particles in a sodium hydroxide solution over time [36]. Copyright 2013 Springer Nature.

Glukhovsky [8] first proposed a model based on the alkali-activation reaction of aluminosilicates in the 1950s, and divided the reaction into three main stages: “alkali solubilization—condensation—polymerization”. As shown in Figure 1, the silicate material dissolves under alkaline conditions and subsequently produces aluminum–oxygen tetrahedron and silicon–oxygen tetrahedron that undergo condensation reactions to form a gel system, and finally, the gel undergoes polymerization reactions. Subsequently, when the concept of geopolymers was introduced by Professor Davidovits [2,31] in the 1970s, his proposal was accompanied by a two-stage “depolymerization-condensation” reaction mechanism for geopolymers. Davidovits considered aluminum–oxygen tetrahedron and inorganic silica–oxygen tetrahedron as the basic units of the composition of geopolymers. The silica–aluminum substances undergo depolymerization and polycondensation reactions through exciters, which eventually form a three-dimensional mesh-like inorganic gel material with no fixed shape. With the development of modern instruments and technologies, microstructure and nanostructure studies of fly ash alkali-activation reaction products (geopolymers) could be carried out utilizing XRD, SEM/EDX, and FTIR, so that the corresponding theoretical systems could be established [32,33]. The structural changes in fly ash geopolymer in various periods were studied by SEM and TEM characterization methods, and a descriptive model of microstructural development of fly ash-based cementitious geopolymer was established [34]. Characterization of fly ash from different sources by XRD and FTIR techniques enables us to study the effect of phase composition on the dissolution behavior, reactivity and finally physical and mechanical properties of fly ash-based polymeric materials [35].

Peter Duxson et al. [37] proposed a mechanistic model to explain the reduced structural reorganization and densification in the microstructure of ground polymer gels in activated solutions containing high concentrations of soluble silica. This work has further demonstrated the “depolymerization-condensation” reaction mechanism proposed by Davidovits. The current models of geopolymer reaction mechanisms are based on such theory, which has gradually formed a complete system after many years of research [38].

### 2.2. Status of Geopolymer Research

**Figure 2:**
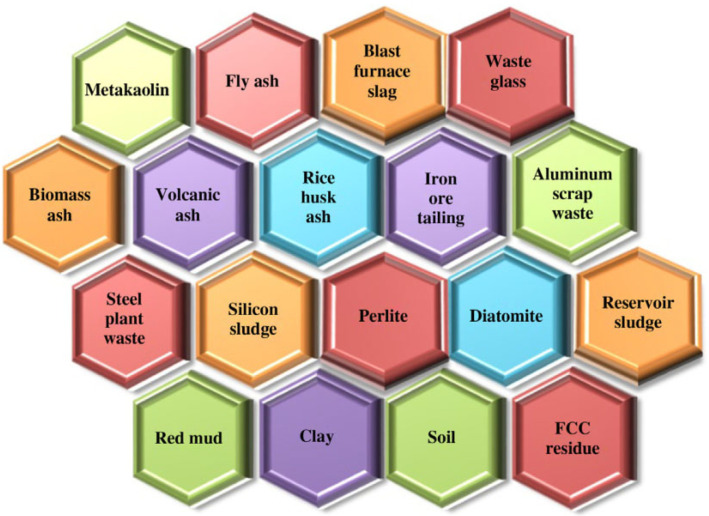
Source of primary material for the preparation of geopolymers [41]. Copyright 2020 Elsevier.

With the advancement of research and the development of science and technology, a fundamental theoretical system of any scale has been formed for several research directions, such as the composition, microstructure, mechanical properties and reaction conditions of geopolymers. P. Duxson et al. [39] summarized the history and review of geopolymer technology in 2006, which focused on the basic chemical structural properties of geopolymers derived from three raw materials: metakaolin, fly ash and slag. Analyzing the influence of processing conditions as effectively as raw materials in the chemical and physical properties of geopolymer products, based on three materials, the survey results show that specific interactions occurred in different systems. Various kinds of aluminosilicate materials are commonly used as raw materials for the preparation of geopolymers, and industrial solid wastes rich in silica and alumina as shown in Figure 2. The differences in mineral composition, morphology, fineness, glassy phase content and chemical properties of raw materials eventually lead to significant differences in the characteristics of geopolymer products [40].

Structural differences in performance result in different properties, and micro differences are factors that affect geopolymer capabilities. Microstructure and mechanical development of the cementitious materials produced by fly ash alkali activation is very significantly influenced by soluble silica, and the percentage and composition of the reaction products depend on the concentration of soluble silica in the activation solution and the period of thermal solidification [42]. Different proportions of fly ash-based polymer concrete with silica fume were fabricated with NaOH/Na_2_SiO_3_ for silicate concrete. The researches revealed that the addition of silica powder increased the compressive strength of geopolymer concrete, and the tensile and flexural strengths were increased with the increase in silica content, indicating that the ratio of SiO_2_/Na_2_O plays a crucial and significant function in the excellent performance of geopolymer [43]. The content of water, sodium hydroxide and sodium silicate during the synthesis of fly ash-based polymers has a remarkable effect on their compressive strength. After a series of characterization and performance tests, the compressive strength was found to increase with decreasing water content and increasing sodium silicate content during the synthesis of the geopolymer, showing a positive correlation between the two [44]. The original materials of geopolymers differ in structure, components and properties, resulting in obvious differences in mechanical and chemical properties of the final products. Three phosphate-based polymer blends were designed and synthesized using silica powder, metakaolin and mono-aluminum phosphate as raw materials. When the Al/P molar ratio was 1.0, the hardened ground polymer reached a compressive strength of about 31 MPa at 28 days [45]. The geopolymer compatibility design and mechanical properties are inseparably related, and both play a mutual role in determining each other. In practical production applications, the ratios of experiments are designed according to the requirements. Proper geopolymer ratio design could be obtained for the required mechanical properties, physical structure and chemical properties [46].

For the preparation of geopolymer materials, activators play a crucial function. Si/Al-rich metakaolin and fly ash form three-dimensional structures under the catalytic effect of exciters, which makes geopolymer activators a focus of research [36]. Priyadharshini Perumal [41] summarized the effect of commonly used alkali activator sodium hydroxide (NaOH) and potassium hydroxide (KOH) on the properties of porous geopolymer materials and found through numerous scientific studies that the amount of alkali is critical for porosity. It became apparent that the compressive strength of phosphoric acid-based polymer blocks of cement ranged from 36.4 to 93.8 MPa, increasing with the concentration of phosphoric acid solution [47]. It was indicated that phosphoric acid (H_3_PO_4_) could be used as an activator instead of sodium hydroxide (NaOH)/potassium hydroxide (KOH).

Salt activators are mild and require calcination at different high temperatures or other pretreatments to improve their reactivity as geopolymer precursors. For example, zeolite is calcined at high temperatures, and when the zeolite structure is destroyed, “meta-zeolite” is obtained, and potassium silicate solution is subsequently applied to activate the meta-zeolite to obtain the geopolymer [48]. Research demonstrates that alkali, acid and salt could be activations agents, but alkali activation agent research earlier, the technology is more mature, while acid activation agent and salt activation agent research studies are fewer. Regarding the current research results suggest that the most effective of the three types of alkaline excitants, and acidic excitant has high requirements for acidity, salt activation effect is ineffective, and the ingredients require activation treatment or mixed with acid/alkali excitant, resulting in higher costs after the application of salt excitant. Activators affect the porosity, bulk density, apparent density, water absorption, etc. of geopolymers, thus affecting flexural and compressive strengths [49]. This suggests that the species and the amount of activator determine to a certain extent the physical and mechanical properties of the geopolymer, and that changes in structure eventually result in changes in chemical properties.

**Figure 3:**
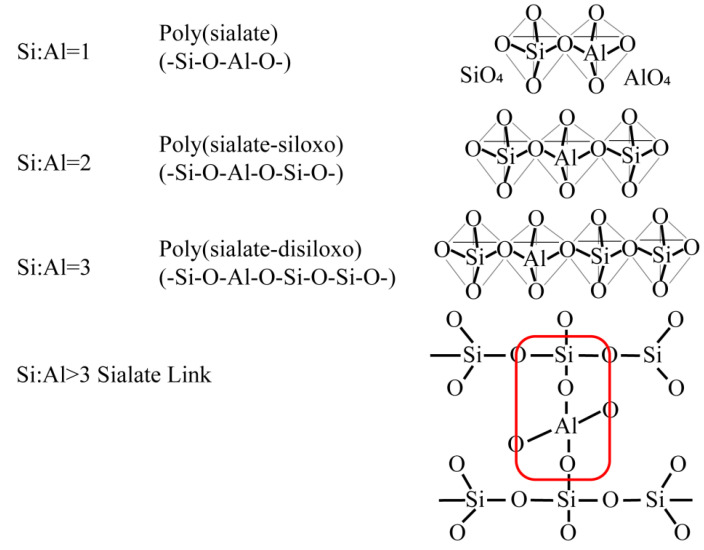
Geopolymer structural system based on the number of silicone Si–O units. Adapted with permission from Ref. [38].

A series of studies have indicated that there is a complex relationship between the properties of geopolymer materials and many conditions, such as the type of raw material, preparation conditions, type of activator, and the ratio of each element [50,51,52,53]. With the expansion and depth of experimental research, the degree of influence of each factor on the performance of geopolymer materials and the influence relationship is gradually being explored. The obvious changes in compressive strength, among other factors, reflect directly the influence of various factors on geopolymer properties. Most obvious among the influencing factors is that silicon content has a positive correlation with strength and hardness, i.e., the bending strength and compressive strength of the material increase with the increase in silicon content, probably because of the different ratios between Si and Al, which eventually lead to different structures of the substance, thus affecting the physical and chemical properties of the geopolymer. The irregular reaction between Si and Al leads to it having a three-dimensional Si–O–Al polymeric network ranging from amorphous to semi-crystalline as shown in Figure 3 [38]. Research scholars have continuously tried to combine the influencing factors in experiments to test the performance, study morphology and investigate the reaction mechanism involved in the reaction process to achieve the goal of preparing green, economical, and cost-effective geopolymer materials with excellent performance.

### 2.3. Application of Geopolymers

**Figure 4:**
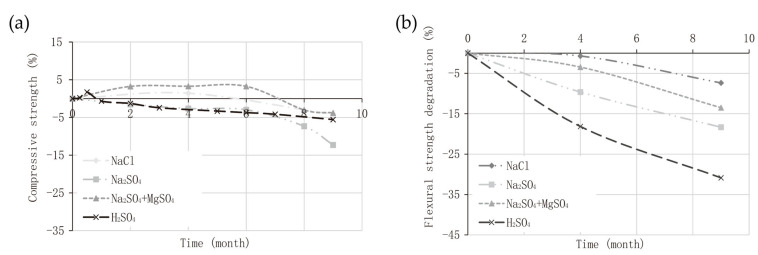
(**a**) Compressive strength degradation of granulated lead smelter slag geopolymer exposed to different chemicals; (**b**) Decrease in flexural tensile strength of granulated lead smelter slag geopolymer [58]. Copyright 2020 Elsevier.

Geopolymers were originally applied in the construction industry, and researchers have reported a typical reduction in CO_2_ emissions of 55–75% for geopolymer concrete versus ordinary silicate cement (OPC) [54,55,56]. As shown in Figure 4, geopolymer concrete has strong acid and alkali resistance, and has better flexural tensile strength than ordinary concrete. Compared to OPC concrete, geopolymer concrete has slightly higher tensile and durability effects as a building material and provides various properties such as superior heat and cold resistance [57,58,59]. Geopolymer concrete could decrease energy consumption, reduce the production cost of construction materials, minimize greenhouse gas emissions and thus decrease the pressure on environmental protection [60,61].

With its unique structure and excellent performance, geopolymer has broad application prospects: noise isolation could use a nanostructured geopolymer, which provides better noise reduction than conventional concrete [62]; innovative super capacitors prepared with graphene and geopolymers that demonstrate improved mechanical and electrochemical properties [63]; preparation of geopolymer-based protective coatings (GPCs) by geopolymers [64]; absorption of heavy metal pollutants from water [65]; preparation of multiphase catalysts [66]. After processing, the geopolymer could directly be treated as structural material, but also as a material carrier for other substances to improve the performance of the original material, such as enhancing the compressive strength, bending strength, acid and alkali resistance of the original material. Immobilization of carbonic anhydrase on geopolymer microspheres significantly improves the performance of carbonic anhydrase and enhances carbon dioxide capture and sequestration by immobilizing the enzyme [67]. In addition, geopolymer-biomass mesoporous carbon composites with mesoporous and macroporous structures are used for solar energy harvesting and could efficiently generate water vapor when used in conjunction with corresponding devices [68]. Various reports have demonstrated that geopolymers are extremely adaptable depending on their structure and could be used in combination with a variety of substances to meet different demands. The low economic cost and the simple preparation process make geopolymers the most promising innovative inorganic porous materials of modern society in the fields of construction, metallurgy, environmental management, fire protection, civil engineering, aviation, soil remediation and catalysis [66,69,70,71,72].

## 3. Application of Geopolymers in Adsorption

**Figure 5:**
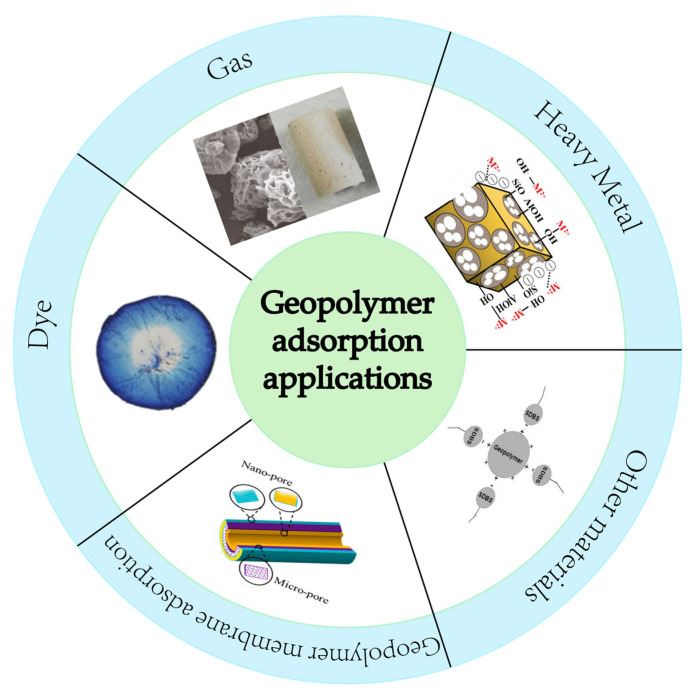
Application of geopolymer adsorption: Other materials [75]; Copyright 2019 Elsevier. Geopolymer membrane adsorption [76]; Copyright 2021 Elsevier. Dye [77]; Copyright 2020 Elsevier. Gas [78]; Copyright 2019 Elsevier. Heavy Metal [79]; Copyright 2019 Elsevier.

Geopolymer has a three-dimensional mesh structure that provides the geopolymer with high porosity and a significant number of mesopores that enhance the adsorption capacity by providing more exposed binding sites on the surface [73,74]. The high mesoporous structure, high porosity, and three-dimensional mesh structure give geopolymers a larger specific surface area, which increases the contact sites with pollutants and impurities. The strong adsorption performance and the high contact sites make geopolymers unique and advantageous in adsorption applications as shown in Figure 5.

### 3.1. Heavy Metal

**Figure 6:**
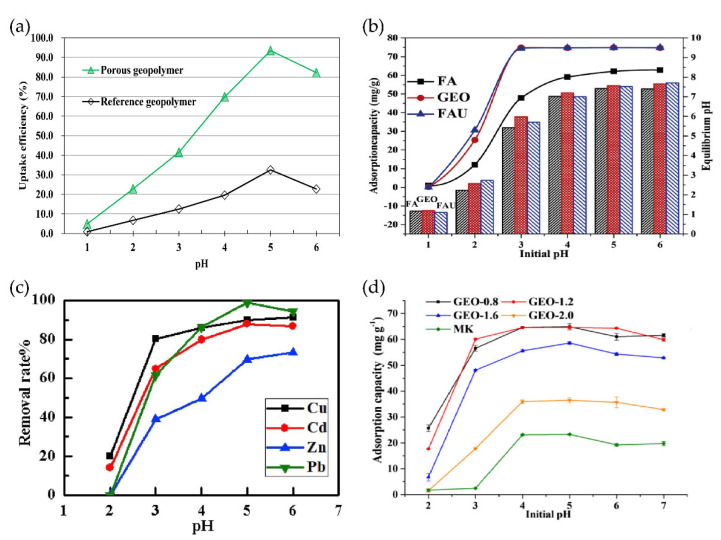
Effect of pH on the adsorption capacity of geopolymers: (**a**) Porous geopolymer and reference geopolymer on the adsorption capacity for Cu^2+^ [82]; Copyright 2016 Elsevier. (**b**) Fly ash (FA), geopolymer (GEO) and faujasite block (FAU) on the adsorption capacity for Pb^2+^ [84]; Copyright 2016 Elsevier. (**c**) Adsorption capacity of Cu^2+^, Cd^2+^, Zn^2+^, pb^2+^ by hollow gangue microsphere/geopolymer [79]; Copyright 2019 Elsevier. (**d**) Adsorption capacity of Cd^2+^ by metakaolin geopolymers [85]. Copyright 2019 Springer Nature.

Water sources in several worldwide suffer from heavy metal pollution, while geopolymers with their strong ion exchange capacity [80,81] and three-dimensional structure become potential materials for heavy metal adsorption. The preparation of adsorbent materials using geopolymers as source material for research on adsorption/immobilization of metal ions has been carried out extensively until now. The adsorption experiments of Cu^2+^ using fly ash and iron ore tailing to synthesize porous amorphous geopolymer revealed a total porosity of 74.6%. The uptake capacity reached the highest value of 113.41 mg/g at 40 °C [82]. It is remarkable that when geopolymer is used as an adsorbent, pH has a significant effect on the adsorption capacity of most of the adsorbents, and pH regulation is often required to obtain the best adsorption effect. As shown in Figure 6, when the pH is low, the solution contains high amounts of H_3_O^+^, which competes with metal cations for the exposed active sites of porous geopolymers, resulting in a lower capacity to adsorb cations such as Cu^2+^, Zn^2+^, Pb^2+^, etc. When the pH increases, H_3_O^+^ decreases, and the competition for metal cations becomes stronger. Take Cu^2+^ as an example, when the pH value is high, the OH^-^ concentration in water increases, and a large amount of OH^-^ generates Cu(OH)_2_ precipitation with Cu^2+^, resulting in poor adsorption performance. The maximum monolayer adsorption capacities were 72.3 mg/g and 69.2 mg/g for Mn^2+^ and Co^2+^ for the removal of Mn^2+^ and Co^2+^ heavy metal ions from aqueous solutions using metakaolin-based polymers. The adsorption efficiencies in this test were observed to be slightly sensitive to temperature and ionic strength, but high adsorption rates could be obtained without pH adjustment [83].

The silicate category of materials itself also has certain adsorption properties. The fly ash, fly ash-based polymer and faujasite block were compared in the experiment and Pb^2+^ was used as the adsorption target. When pH = 3, the maximum adsorption capacities of fly ash, geopolymer and faujasite block were 49.8, 118.6 and 143.3 mg/g, respectively, indicating that the adsorption efficiency of geopolymer and faujasite block was much higher than that of fly ash, and the entropy difference in adsorption among the three indicated that fly ash was mainly physical adsorption of Pb^2+^, while geopolymer and faujasite block are chemisorbed [84]. Metakaolin-based polymers were used as adsorbents for the removal of Zn^2+^ and Ni^2+^ ions from aqueous solutions, and the maximum monolayer adsorption capacities of Zn^2+^ and Ni^2+^ determined by Langmuir adsorption isotherms were 1.14 × 10^−3^ and 7.26 × 10^−4^ mol/g, respectively. Adsorption research in continuous mode demonstrated that the optimal flow rate was 2.0 mL/min for Zn^2+^ and 1.0 mL/min for Ni^2+^ [86].

Under the experimental conditions of continuous operation, it was discovered that besides the common influencing factors, namely, initial concentration of ions, amount of adsorbent, adsorption time, pH, etc., flow rate also affects the adsorption efficiency of the material. The geopolymers prepared at different alkali activator moduli (silica/sodium oxide = 0.8, 1.2, 1.6, 2.0 mol/L) using the same metakaolin raw material had different adsorption capacities when prepared with different alkali activator moduli agents. When the geopolymer prepared with alkali activator modulus of 0.8 mol/L showed the best adsorption performance for Cd^2+^, the maximum adsorption capacity was 70.3 mg/g [85].

To improve the adsorption capacity and various properties of the adsorbent, the geopolymer could be modified and altered by adding other substances during the preparation of the geopolymer. Synthesis of innovative geopolymer–alginate–chitosan complexes using a mixture of metakaolin geopolymer and sodium alginate solution and chitosan for direct removal of Pb^2+^ from wastewater [87]. Hollow gangue microspheres were inserted into the geopolymer matrix by the geopolymer method for the removal of heavy metal ions (Cu^2+^, Cd^2+^, Zn^2+^ and Pb^2+^) from aqueous solutions. The adsorption principle of this adsorbent material was attributed to physical, chemical, electrostatic attraction, and ion exchange by pseudo-second-order kinetic model fitting, equilibrium isotherm adsorption data and Langmuir equation fitting analysis in this experiment [79]. While silicates are generally used for geopolymers, other substances could be used to provide silica and aluminum sources for the preparation of geopolymer-based adsorbent materials. Some nano-zeolite and geopolymer/zeolite products could be synthesized at low cost by using rice husk and waste aluminum cans as silicon and aluminum sources, respectively, and the synthetic products could effectively remove Co^2+^, Cu^2+^ and Zn^2+^ [88].

### 3.2. Other Ions

**Figure 7:**
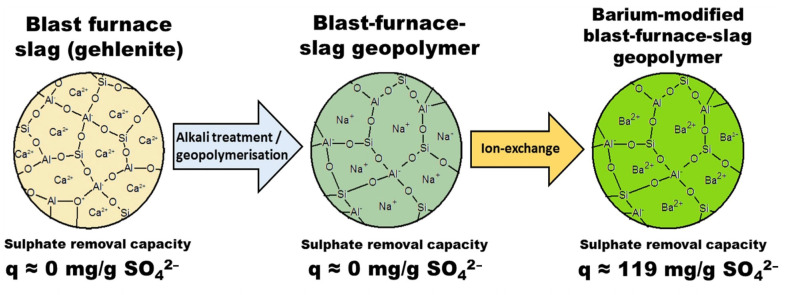
Structure and sulphate removal capacity of blast furnace slag (gehlenite), blast-furnace-slag geopolymer and barium-modified blast-furnace-slag geopolymer [92]. Copyright 2016 Springer Nature.

Geopolymer adsorbent materials could adsorb NH_4_^+^, anionic sodium dodecyl benzene sulfonate and other pollutants, in addition to common heavy metal ions. The geopolymer of metakaolin was made into granular form for the adsorption of NH_4_^+^. Experiments were conducted in municipal wastewater treatment plants on a pilot basis, and the NH_4_^+^ effluent concentration was always maintained at 4 mg/L at a water temperature of 10 °C [89]. The application of geopolymer adsorbent materials for NH_4_^+^ removal from waste leachate and NH_4_^+^ removal from piggery wastewater has also been investigated [90,91]. This work indicates that the porous ground polymer particles have high selectivity for NH_4_^+^ even in the presence of organic compounds and competing ions [91]. The geopolymer adsorbent could capture the anionic surfactant sodium dodecyl benzene sulfonate, similarly to the above-mentioned adsorption process, which the adsorption is chemical and physical at the same time [75]. Cation exchange processes (Na^+^ substitution by Ba^2+^) occur in the framework structure of the geopolymer using Ba for geopolymer modification. The Ba-modified geopolymer materials exhibited higher adsorption rates for SO_4_^2−^ in the pH range 7–8, as shown in Figure 7 [92].

Adsorption calculations by pseudo-second-order kinetics, Langmuir isotherm model, etc. suggested a uniform distribution of adsorption sites and the formation of a monolayer of adsorbate on the surface of the geopolymer in most of the adsorption processes. In several studies, heavy metal ions are better adsorbed under strongly acidic conditions because the solution contains more H^+^ in strongly acidic conditions, which means that there is the higher free energy of hydration and could exchange metal ions more effectively [93]. The adsorption capacity is not limited to the adsorbent material itself but is determined by the experimental conditions. Different experimental conditions have to be explored for different ions in return for the best adsorption effect.

### 3.3. Dyes

**Figure 8:**
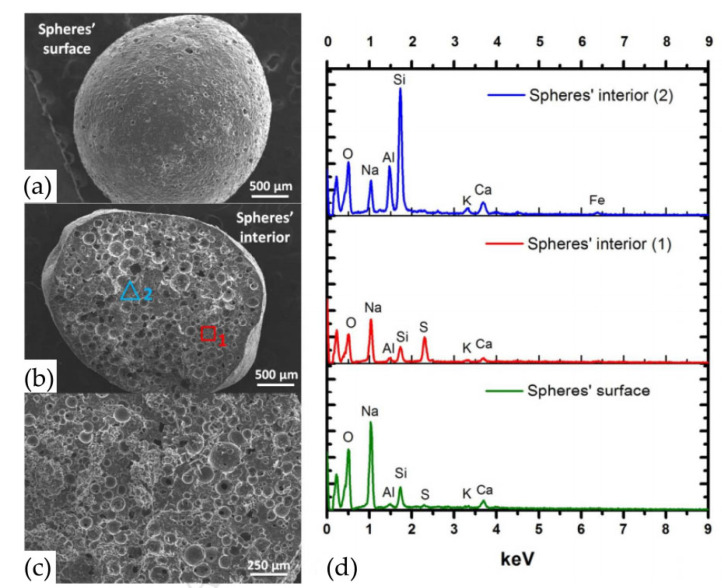
SEM micrographs of the geopolymer spheres: (**a**) surface and (**b**,**c**) interior; (**d**) presents the EDS spectra of the geopolymer spheres surface and interior (at two different positions) [94]. Copyright 2018 Elsevier.

In research on the extraction of methylene blue from synthetic wastewater by fly ash-based geopolymer spheres, the removal efficiency of methylene blue uptake reached 79.7 mg/g and remained up to 83% after eight cycles of regeneration. The surface of the spheres is smooth, as shown in the SEM of Figure 8, and the large number of pores inside the spheres, and the open small pores on the closed macropores expose more active sites to improve the adsorption. Additionally, the significant difference in chemical elements between two parts of the spheres was found by EDS, which was caused by the inhomogeneity of the particles and the chemical differences in the precursors [94].

The geopolymer balls could be prepared by liquid nitrogen drop technique, in which PEG600 is added as a binder to adsorb methylene blue, and the removal efficiency of all beads after 24 h reaches 98% removal on average. The efficiency observed in this study is mainly associated with the morphology and porosity of the beads, which in turn is directly related to the water content added to the geopolymer slurry [77]. Other research on the adsorption of methylene blue has demonstrated that this adsorption method is effective and feasible, and is expected to be used directly in industrial wastewater treatment in the future [89,95,96]. These fly ash-derived inorganic polymers also exhibit excellent adsorption properties when adsorbing crystalline violet from aqueous solutions [97]. Magnetic geopolymers were used as effective aqueous decolorization adsorbents for the removal of acid green (AG) and procion red (PR) from aqueous solutions. The results of the theoretical calculations of the bilayer adsorption model and the corresponding performance tests confirmed that the active sites of the magnetic ground polymers are favorable for binding to the smallest dye molecules, thus improving the adsorption capacity [98]. Simplified and low-cost surface modification of previously prepared fly ash-based polymers with cetyltrimethylammonium bromide (CTAB) was able to remove anionic acid blue 185 (AB185) without strong acid conditions, with a maximum removal efficiency of 98.2%, and this research indicates that electrostatic interactions also have a major effect on the adsorption process [99]. The mesoporous geopolymer was synthesized using a new and simple synthesis method using metakaolin and rice husk ash as the source of silica and alumina, and the material was tested for the removal of methyl violet 10B (MV10B) from an aqueous solution. The results demonstrated that the maximum adsorption capacity of the mesoporous geopolymer was 276.9 mg/g, and that the adsorption was spontaneously generated with a heat absorption process [100]. With Rhodamine B as the removal target, using geopolymers as adsorbents, the removal capacity was increased as the contact time increased, allowing the active sites to interact more fully with the dye molecules. However, this experiment also showed that when the contact time exceeded a certain critical time, the adsorption capacity stopped increasing [101]. Activated geopolymer with a specific surface area of 35.7 m^2^/g was used for the adsorption of methyl orange (MO) dye, but the experimental results showed that compared to kaolinite and metakaolinite, activated geopolymer had the largest specific surface area, but the adsorption effect was lower than the other two substances. This might be explained by the fact that MO is an anionic acid dye and the silicone bonds on the surface of geopolymer are negatively charged, MO and hydroxyl ions (OH^-^) compete with each other at the adsorption sites [102]. Table 1 summarizes some of the recent studies related to the adsorption of dyes using geopolymers.

**Figure 9:**
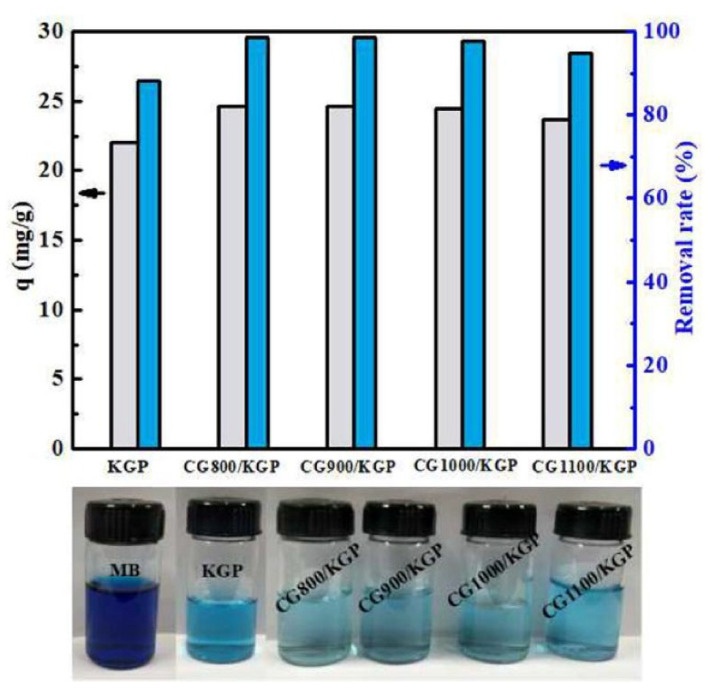
Effect of pre-calcination temperature on the adsorption capacity and removal efficiency of MB on coal gangue microsphere/geopolymer (CG/KGP) samples [96]. Copyright 2018 John Wiley and Sons.

Dye wastewater is expensive to treat because it contains a large amount of pigments and organic matter, while geopolymers possess the characteristics of low cost, green and high adsorption efficiency. The geopolymer adsorbent materials prepared at different temperatures all showed excellent adsorption performance in the treatment of dye wastewater as shown in Figure 9, but geopolymers have poor adsorption performance for both negatively charged ions and anionic dyes, which is caused by the charge characteristics of the geopolymer surface. In a highly acidic environment, the surface of the silica–aluminate mesh of the geopolymer is protonated, thus becoming a positively charged adsorbent on the surface, which facilitates the adsorption of anionic dyes [108]. After modifying the pores, hydrophobicity of the material, surface charge and ionic affinity by adding suitable additives and changing the functional groups on the surface of geopolymers and their crystalline shape, it is expected to be used in large-scale industrial applications for the treatment of dye wastewater [110].

Absorption of heavy metals and dyes on geopolymers is mainly a spontaneous, heat-absorbing and entropy-driven process, and the adsorption process contains physical, chemisorption, and ion-exchange adsorption. Adsorption is frequently the result of the combined effect of three kinds of adsorption, which accounts for the major type of adsorption in the adsorption process as a result of differences in adsorbent substances, adsorption conditions, and adsorbents. Research in the future should focus on: improving the mechanical properties, adsorption performance and stability of geopolymer adsorbent materials; improving the adaptability of geopolymer adsorbent materials under different conditions, such as strong acidic and alkaline environments, organic–inorganic mixed solutions, high temperature and high pressure, and other complex environments; and studying the ability to adsorb pollutants other than heavy metals and dyes, and the application of continuous operation in practical wastewater [110].

### 3.4. Other Adsorption Applications

**Figure 10:**
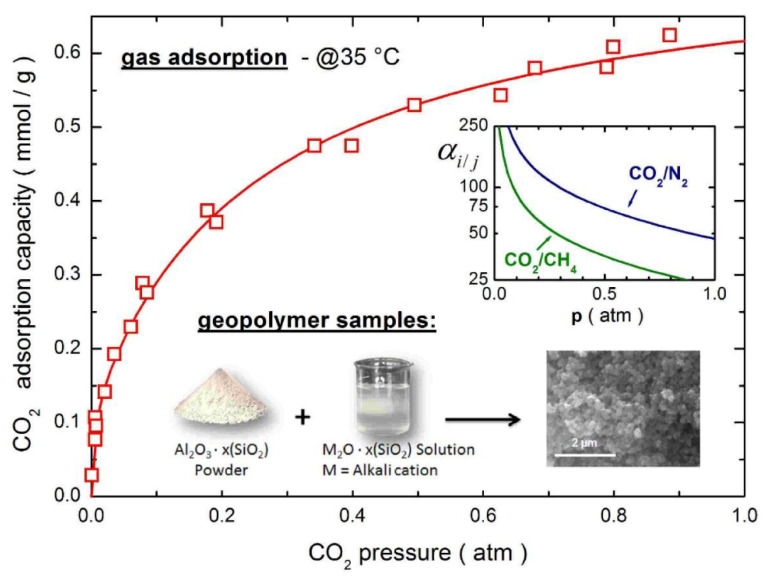
Simple preparation process of geopolymer and its adsorption ability on CO_2_, separation effect of geopolymer on CO_2_/N_2_, CO_2_/CH_4_ gas mixture [112]. Copyright 2016 Elsevier.

In recent years, research scholars have focused their attention on the application of geopolymers for gas adsorption. Cryogenic adsorption of CO_2_ in solid materials is a highly cost-effective method for implementing decarbonization in retrofit plant strategies [111]. Selected alkali-based porous ceramic clay polymer materials applied to CO_2_ adsorption exhibit excellent performance for better separation of CO_2_/N_2_ and CO_2_/CH_4_ gases, as shown in Figure 10 [112]. To purpose of increasing CO_2_ capacity, a novel composite material formed by embedding Na13X zeolite in a geopolymer matrix using K/Na silicate material as raw material. the CO_2_ capacity of the Na^+^-based composite is significantly greater than that of the K^+^-based adsorbent (2–3 times) [113]. Simplifying the experimental procedure, a complex three-dimensional pore network NaX nano-zeolite-geopolymer monomer was finally obtained using a one-pot hydrothermal synthesis method, which has a BET-specific surface area of 350 m^2^/g [114]. Practical production in industry produces CO_2_ at high temperatures and mostly mixed gases, requiring adsorbent materials with high mass transfer, strong mechanical properties, great thermal stability and chemical stability. The talc-based powder was mixed with a metakaolin-based polymer matrix to prepare an innovative ground polymer-hydrotalcite composite material, which has excellent compressive strength at temperature and 500 °C while ensuring adsorption capacity, ranging from 10 to 35 MPa [78].

Geopolymers have been researched more in CO_2_ gas adsorption, but the application of other gas adsorption has been less studied. The present studies suggest that geopolymer adsorbent materials with proper modification have high mass transfer, high stability and selective adsorption. They have great potential in the treatment of industrial waste gases, and their adsorption applications and adsorption principles for other industrial waste gases such as SO_2_, CO, and H_2_S could be explored in the future.

In addition to the common adsorption of heavy metals, dyes, and gases, geopolymers could be used for material separation using the principle of adsorption. The adsorption of formaldehyde in aqueous solutions using geopolymers, the cation exchange capacity of geopolymers is 2–3 times higher than that of natural zeolites, and the process of formaldehyde removal includes physical adsorption, chemical adsorption and complexation, which may be the reason for the stronger adsorption capacity of geopolymers for formaldehyde [115]. Experimental adsorption desulfurization of oil using geopolymers. The study revealed the presence of Na, Al, and strong acid sites on the surface of the prepared geopolymer. These sites interact with sulfur compounds in heavy gas oils through π–π and acid–base interactions [116].

### 3.5. Application of Geopolymer Membranes in Adsorption

**Figure 11:**
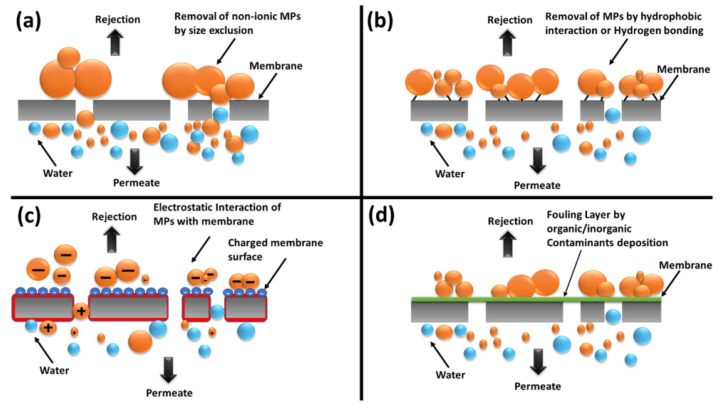
Removal mechanism of membrane purification: (**a**) size exclusion; (**b**) hydrophobicity; (**c**) electrostatic interaction; (**d**) adsorption [118]. Copyright 2019 Elsevier.

Membrane technology has developed rapidly in recent years and is widely used for the separation, classification, purification or enrichment of multi-component liquids or gases as shown in Figure 11. However, most of the mature membrane technologies used nowadays are organic membranes, which have the advantages of high treatment efficiency and easy operation, but have the disadvantages of high economic cost and serious membrane pollution. Inorganic membranes make up for the shortcomings of organic membranes, but inorganic membranes are more expensive to prepare, and the process is not mature. Therefore, the emergence of geopolymer membranes provides an opportunity for the development of inorganic membrane applications [117]. Numerous studies have proven that geopolymers have excellent adsorption properties, and the advantages of geopolymers could be exploited to prepare low-cost and high-efficiency inorganic membranes. Changing the form of a geopolymer used to make it renewable and reusable expands the scope of geopolymer applications in industrial production.

**Figure 12:**
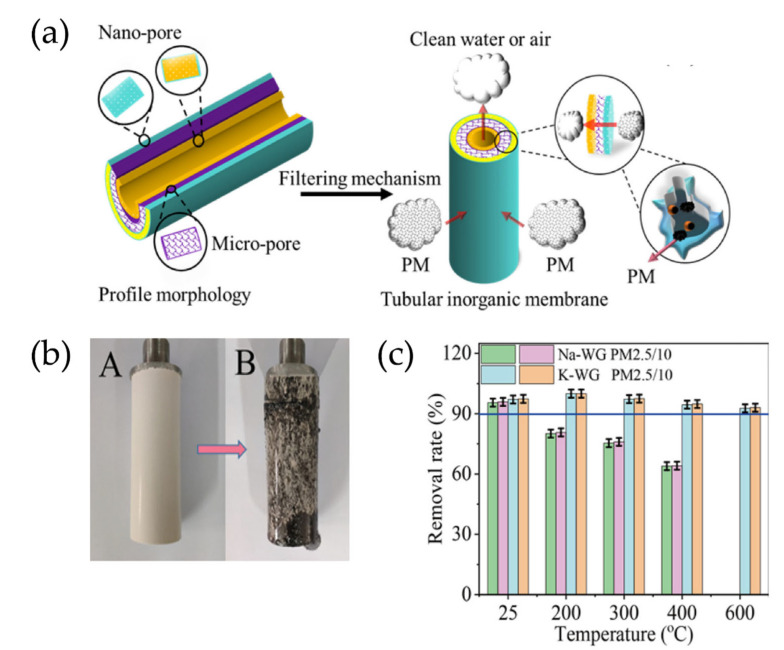
(**a**) Profile morphology of tubular inorganic membrane and the flue gas purification mechanism of particulate matter in inorganic membrane; (**b**) A: inorganic membrane before purification; B: inorganic membrane after purification; (**c**) PM2.5/10 removal efficiency of tubular inorganic membranes at different temperatures [76]. Copyright 2021 Elsevier.

Biomass fly ash ground polymer monomers are used as adsorbents, and these monolithic adsorbents could be used as membranes after being used directly in filled beds [119]. Research has suggested that geopolymers could be prepared directly into membranes. The novel free-sintered self-supporting inorganic membrane simultaneously adsorbs and repels Ni^2+^ during the membrane separation process, which could effectively remove Ni^2+^ from wastewater while removing small molecule pollutants from water [120]. To increase the contact sites, improve removal efficiency, and facilitate use, geopolymers may also be prepared as tubular membranes. Porous gradient geopolymer-based tubular membranes were prepared using a one-step molding method for the removal of particulate matter (PM) [121]. Figure 12 shows the basic principle and treatment effect of a geopolymer tubular inorganic membrane in flue gas treatment.

**Figure 13:**
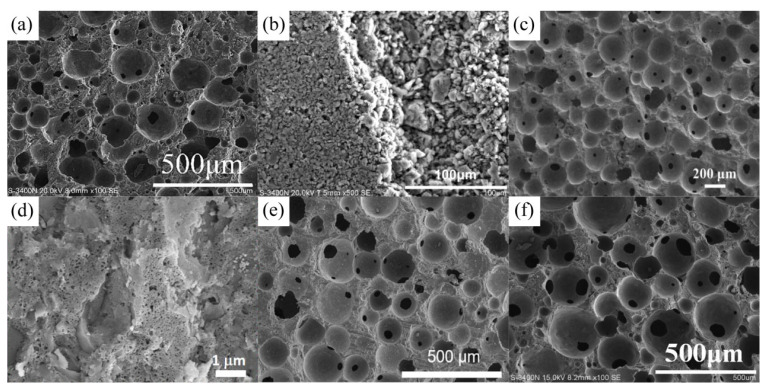
SEM images of different types of geopolymer membrane: (**a**) SEM images of the surface of geopolymer inorganic membrane [123]; Copyright 2018 Elsevier. (**b**) SEM images of the surface and the cross section of geopolymer-based inorganic membrane [120]; Copyright 2015 Elsevier. (**c**) SEM images of the surface of porous zeolite-like geopolymer membrane [124]; Copyright 2020 Elsevier. (**d**) SEM images of cross of the zeolite membrane layer [125]; Copyright 2019 Elsevier. (**e**) SEM images of the surface of the porous geopolymer (PG) [126]; Copyright 2019 Elsevier. (**f**) SEM images of cross of membranes with 0.9 wt% H_2_O_2_ [121]. Copyright 2019 Elsevier.

The geopolymer membrane requires a higher compressive strength while having a greater specific surface area. Besides conventional fly ash or metakaolin, phosphate mine tailings could be used to produce geopolymer membranes, which also exhibit good compressive strength (11.2–43.7 MPa) and high BET surface area (321–384 m^2^/g). The geopolymer membrane could effectively remove Cu^2+^ from water through the combined effect of ground adsorption and repulsion [122]. The SEM in Figure 13 shows that both the directly prepared geopolymer membrane and the modified geopolymer-based membrane have high porosity and could selectively remove the target material from the solution by adjusting the pore size.

Appropriate modification of inorganic membranes could improve their adsorption capacity and mechanical strength. A metakaolin-based polymer composite membrane (support + dense layer) was prepared using metakaolin and alkali solution at lower temperature and atmospheric pressure, using porous ground polymer as a carrier and a dense layer on the surface using a double coating process. The composite membrane exhibited amorphous structure, high permeate water flux (245 kg/m^2^·h), good interface combination and reusability. The membrane performs better on paper wastewater purification, effectively intercepting and adsorbing suspended solids in solution [123]. A new inorganic-organic composite membrane was prepared by electrostatic self-assembly method, choosing porous polymers as the carrier and using the “green” biomaterial chitosan to form the active layer. The effectiveness of the composite membrane in removing crystalline violet (CV) was attributed to the synergistic effect of repulsion and adsorption [126]. The porous zeolite ground polymer membrane (Geo) was used as the immobilization carrier to prepare laccase-immobilized ground polymer composite membrane for CV removal. CV molecules could be adsorbed on the porous Geo surface through the electrostatic attraction effect and van der Waals forces. Secondly, laccase is an oxidoreductase that could oxidize dye molecules, creating a synergistic effect between the two to achieve higher removal efficiency [124].

Geopolymer membranes are convenient to use compared to other forms of geopolymer-based materials and are reusable as they could be backwashed to restore the original flux. Modified and adapted geopolymer membranes have a variety of properties, and while acting as a carrier, the geopolymer membrane’s physical adsorption capacity help adsorb metal ions, resulting in higher removal efficiencies. The combination of geopolymers and other substances could maximize the synergistic effect and maximize the performance of the membrane material.

## 4. Conclusions

Geopolymers are low-cost, environmentally friendly materials with a three-dimensional mesh structure that could have excellent mechanical, thermal and chemical properties by adjusting the ratio, temperature, additives and other preparation conditions. In recent years, researchers have moved beyond the research of single-component geopolymers and have focused on composite geopolymer materials. Compared to single-component geopolymer materials, composite geopolymer materials offer better performance.

The results of a series of investigations indicate that geopolymers have excellent performance in the field of adsorption, with large specific surface area, porous structure, ion exchange properties and certain compressive strength [127]. Considering from the perspective of physical properties, the high porosity of geopolymers allows interception of some pollutants and has a larger specific surface area, exposing more active sites; thinking from the perspective of chemical properties, the modification changes the chemical properties of the material and enhances the adsorption performance. Geopolymers with great room for innovation are expected to be industrially applied in the future in neighborhoods involving adsorption such as wastewater treatment, waste gas treatment, purification, separation and pigment removal.

The best characteristic of geopolymers is that the properties of geopolymers could be adjusted according to the needs of the application, such as modifying the pore size range and surface charge according to the molecular size of the adsorbent; changing the hydrophilicity/hydrophobicity of the material surface by modification with other substances, etc. However, this property is like a double-edged sword, which has too many influencing factors in the preparation and application process, requires high experimental precision in the preparation process, and requires high environmental requirements in the surrounding environment when the material is at its maximum performance. The massive use of alkali activators also consumes resources, resulting in elevated geopolymer emissions and increased costs. The current problems become a great resistance to the practical application of geopolymers. In response to the shortcomings of geopolymers, the following perspectives are proposed:

1. The components of geopolymers have a significant impact on the material properties, and further innovative development of composite geopolymer materials while improving the performance of existing geopolymer materials.
2. Geopolymers are strongly influenced by various factors, and the mechanisms should be explored in depth. For example, how does the ratio of raw materials affect the final formation of geopolymers? How do the coupling agents, K+, Na+ and other added substances affect the performance of geopolymers? By studying the mechanism of each step in depth, a complete and comprehensive theoretical system should be established to turn the uncontrollable process in the experiment into one that is controllable.
3. The raw materials for the present preparation of geopolymers are mainly natural minerals, and the use of pure chemicals for their preparation may be explored. Try to apply geopolymers in other neighborhoods such as fine chemicals, chemical separation, microelectronics, electrical, etc.
4. Geopolymer and zeolite have similar structures. There are few studies on the selectivity of zeolite-based adsorbents, and the selective adsorption of geopolymers should be further studied.
5. For industrial applications, geopolymers could be prepared as flat membranes, tubular membranes, multi-channel membranes, and single or multiple geopolymer membranes assembled into membrane assemblies. Complete membrane units are more conducive to industrial production and laboratory research.
6. Finally, the basic research on geopolymers and their practical application still has enormous research space, from many aspects such as performance, cost, emission and use, to transfer the innovation of geopolymers to practical application based on theoretical knowledge.

## Figures and Tables

**Table 1:** Comparison of the adsorption capacity of geopolymers for different dyes and summary of the specific surface area, total pore volume, and average pore size of geopolymers. Initial concentrations of contaminants (Co), adsorption capacity (q_max_), removal efficiency ®, specific surface area (SSA) are shown.

Geopolymer	Dye	Co (ppm)	q_max_ (mg/g)	R (%)	SSA (m^2^/g)	Total Pore Volume (mm^3^/g)	Average Pore Diameter (nm)	Ref.
G13A-20TiO_2_ geopolymer	MB	50	42	82	20	714	110	[103]
Synthesized geopolymer paste	MB	32	n.r. ^1^	85.6	5.8	n.r.	1300	[95]
CG900/KGP porous particles	MB	100	n.r.	98.5	37.3	352	37.8	[96]
Metakaolin-based geopolymer	MB	60	2.7	81.5	26.4	n.r.	30.3	[104]
G-70-P geopolymer	MB	50	5.1	99	21	1252	320	[77]
Seawater-based geopolymer	MB	n.r.	59.5	81.4	11.9	25	25	[105]
CTAB-modified fly ash-based powder	AB 185	30	0.2	96.8	34.9	138	11.7	[99]
Magnetic geopolymer	AG	n.r.	n.r.	n.r.	19.5	45.3	10.4	[98]
Magnetic geopolymer	PR	n.r.	n.r.	n.r.	19.5	45.3	10.4	[98]
Magnetic geopolymer	AG 16	50	400	85.9	53.4	190	14.1	[106]
Magnetic geopolymer	Acid Red 97	100	1814.3	97	42.9	52.3	4.9	[107]
MGP geopolymer	MV10B	50	21.6	73.8	62	360	14.3	[100]
Geopolymer lattice with activated carbon (3D Printing)	Orange II	400	66.5	n.r.	185.6	n.r.	n.r.	[108]
Alkaline-activated geopolymer	Eriochrome Black T	40	251	98	74.5	60	3.2	[109]

^1^ n.r. = not reported.

## Data Availability

The data presented in this study are available on request from the corresponding author.
